# Emergence of methicillin resistant *Staphylococcus pseudintermedius* in dogs sampled in 2018 in the island nation of Grenada, West Indies

**DOI:** 10.3389/fvets.2026.1761713

**Published:** 2026-03-18

**Authors:** Josephine A. Afema, Cristina Mastromonaco, Margaret A. Davis, Diana M. Stone, Lisa P. Jones, Tara E. Paterson, Marta L. Perea, Brian P. Butler

**Affiliations:** 1 Department of Pathobiology, School of Veterinary Medicine, Saint George's University, Saint George's, Grenada; 2 Paul G. Allen School for Global Animal Health, College of Veterinary Medicine, Washington State University, Pullman, WA, United States; 3 Department of Small Animal Medicine and Surgery, School of Veterinary Medicine, Saint George's University, Saint George's, Grenada

**Keywords:** antimicrobial resistance (AMR), environmental contamination, Grenada, methicillin resistant *Staphylococcus pseudintermedius* (MRSP), canine

## Abstract

*Staphylococcus pseudintermedius* (SP) is a commensal bacterium, and an opportunistic pathogen of dogs. Emergence and global dissemination of methicillin resistant SP (MRSP), environmental contamination in veterinary clinical settings, and nosocomial infections are of concern to veterinary medicine. Furthermore, zoonotic infections impact public health. By 2014, only methicillin susceptible SP were detected in canine pyoderma cases in Grenada. We hypothesized MRSP might be introduced to Grenadian dogs via international movement of dogs owned by foreigners associated with St. George's University (SGU). We compared antimicrobial resistance patterns, and genotypes in SP from foreign-born and Grenadian-born dogs. Oral and perianal swabs were collected from 85 dogs owned by SGU faculty, staff, students, and SGU security dogs. Skin swabs were obtained from 25 of these dogs with cutaneous lesions consistent with pyoderma. Bacteria were isolated and identified using culture, PCR, and MALDI-TOF. Antimicrobial susceptibility testing was performed using disc diffusion assay, and genotypes determined by multi-locus variable number of tandem repeat analysis. A questionnaire was administered to investigate risk factors for MRSP infection. Dogs were classified as foreign-born (*n* = 58) or Grenadian-born (*n* = 27). Overall, MRSP prevalence was 9.4%, with no difference between foreign-born and Grenadian-born dogs. Univariable analysis indicated that antimicrobial use was associated with MRSP infection, however, other factors e.g. age, sex, hospitalization or country/region of birth were not. *Staphylococcus pseudintermedius* was isolated from 37.4% (73/195) of dog samples, and from 11.9% (10/84) of the environmental samples. Methicillin resistant SP constituted 13.3% of SP isolates, and MRSP was documented for the first time in Grenadian dogs. Detection of a similar MRSP strain in foreign-born and Grenadian born dogs suggests transmission occurred between these dogs. Genotypic, resistance, and epidemiologic data could be consistent with reports of MRSP dissemination via dog movements. Detection of a similar MRSP strain in foreign-born dogs and animal contact surfaces in the Small Animal Clinic supports the plausibility of environmental contamination and nosocomial transmission, and highlights the importance of disinfection procedures. Compared to the 2014 study, the frequency of resistance to most antimicrobials increased. Monitoring trends in antimicrobial resistance is important for making informed animal health decisions.

## 1 Introduction

*Staphylococcus pseudintermedius* (SP) is a commensal bacterium that resides on the skin and mucosae of dogs where it is host-adapted. It is also an opportunistic pathogen that is associated with various clinical conditions such as pyoderma, surgical wound complications, otitis, and urinary tract infections (1). The emergence and global dissemination of methicillin resistant *S. pseudintermedius* (MRSP) lineages is a major threat to animal health (2). Methicillin resistant SP are a type of SP that are resistant to methicillin and other antimicrobials in the beta-lactam class. Beta lactams are critically important for treating infections in human and veterinary medicine. In staphylococci, methicillin resistance is primarily mediated by the *mecA* gene, which encodes penicillin-binding protein 2a (PBP-2a) and confers resistance to beta-lactam antimicrobials (3). The *mecA* gene was detected in staphylococci from dogs for the first time in 1999 (4). Since 2006, methicillin resistant staphylococci, and especially MRSP strains have emerged and been detected in dogs globally (1). Methicillin resistant SP strains often carry additional resistance to other classes of antimicrobials, hence, infections can be difficult to treat and may recur (1, 5, 6).

Several risk factors including hospitalization, frequent hospital visits, and chronic skin conditions have been associated with MRSP infections in dogs and cats (7). For instance, infections and disease outbreaks have been attributed to contaminated hospital environments and contaminated or colonized hospital staff and this may provide opportunities for dissemination of MRSP to dogs (8). Notably, MRSP can persist for several months in patients that receive repeated treatments for recurrent infections (7). Such patients might contaminate hospital environments during repeat visits. Furthermore, the presence of MRSP in a small animal hospital environment despite implementation of routine cleaning and disinfection procedures complicates infection prevention and control in hospital settings (9).

Evaluation of trends in antimicrobial resistance (AMR) levels in SP from healthy and clinically affected dogs can uncover new resistance patterns, reveal changes in resistance levels, and provide useful information on antimicrobials that are effective (10–14). For instance, a review of AMR trends in clinical and commensal SP and *S. intermedius* of canine origin from 27 countries for the period 1980–2013 did not find a significant increase in resistance to most antimicrobials over time, except resistance to penicillin and ampicillin (11). However, other studies have reported increasing frequency of resistance to highly and critically important antimicrobials among SP and other *Staphylococcus* spp. obtained from dogs (10, 15). From 1999–2014, there was a significant increase in resistance to most antimicrobials among clinical staphylococci isolated from dogs in Portugal (11). Also, in Canada, SP obtained from healthy dogs in 2014 displayed greater frequency of resistance to most antimicrobials, and multidrug resistance compared to a 2008 study (15).

Evaluation of the global epidemiology of SP isolates of canine origin indicate SP has diverse genetic backgrounds and there are differences in the population structure and antimicrobial resistances between methicillin susceptible SP (MSSP) and MRSP isolates (16). Certain MRSP lineages are associated with specific resistance patterns and have undergone clonal expansion and dissemination over wide geographic distances, whereas predominant clones have not been detected among MSSP isolates (5, 6, 16). Characterization of MRSP isolates obtained in the period between 2004 and 2009 from diseased and healthy dogs across several countries found the predominant clonal lineage in Europe belonged to ST71, while the predominant clonal MRSP lineage in North America belonged to ST68 (5). A subsequent study reported that MRSP clonal lineage associated with ST71 had become more widely spread across the globe (17, 18). The epidemiology of MRSP continues to evolve with emergence of new MRSP clonal lineages and replacement of formerly prevalent clones (17). The MRSP clonal lineages that are in circulation in dogs in the Caribbean are mostly unknown, apart from a study in Trinidad and Tobago that found MRSP ST71 in one dog and MRSP ST45 in another dog (19).

To our knowledge, the only study on staphylococcal infections in dogs in Grenada was published in 2014, and that study investigated causes of pyoderma and found MSSP but not MRSP (20). Within the Caribbean, there are two reports from Trinidad and Tobago of MRSP in dogs, one from a dog with otitis (21) and another involving healthy dogs (19). The global dissemination of certain MRSP clones has partly been attributed to international movement of dogs (16, 22). In Grenada, foreign faculty, staff and students associated with St. George's University (SGU) often travel with their dogs to the island. We therefore hypothesized MRSP could be introduced into the Grenadian dog population by foreign-born dogs that have been exposed to MRSP in other geographical regions. This study compared genotypes and antimicrobial resistance phenotypes in SP from Grenadian-born dogs and foreign-born dogs, and coupled that with epidemiological data to identify potential transmission events. We also investigated environmental contamination at the SGU Small Animal Clinic.

## 2 Materials and methods

### 2.1 Sample collection

This study was conducted in dogs residing in the Caribbean island of Grenada, West Indies, and sample collection was performed at SGU, School of Veterinary Medicine (SVM). This university attracts international faculty, staff and students from North America, Europe, and other regions, and many of these individuals travel with their pets and/or adopt local dogs that they transport to and from their country. This study therefore targeted pet dogs owned by SGU faculty, staff and students and SGU security dogs. Study animals were recruited between February and May 2018 by sending emails and posting information on Facebook and notice boards on the SGU campus. Sample size was estimated using a formula for cross sectional studies, assuming MRSP prevalence of 2.5%, 2.5% margin of error, and 95% confidence level (23). Dog owners that agreed to participate in the study brought their dogs to the SGU campus for sample collection. A signed consent was obtained, then a clinician performed a physical examination and obtained an oral swab and a perianal swab. The oral swab was obtained by inserting a swab into the buccal cavity between the cheek and teeth and rotating it at least three times. The perianal swab was obtained by swabbing around and across the anal area. These sites were chosen because they are frequently colonized and are sensitive for detection of SP (12, 15). For dogs with cutaneous lesions or external ear infections, additional swabs were obtained from the lesions.

A questionnaire was designed to examine risk factors for SP or MRSP infection (Supplementary File 1). Data obtained included age, sex, country of birth, travel history, medical history (general health, treatments, hospital visits) and social behavior (interaction with other dogs). The questionnaire was administered to dog owners before sample collection. Participants took about 10–15 min to complete the questionnaire and a researcher was available to answer any questions. Most questions had dichotomous responses, and space was provided to explain responses. Some questions were unstructured or multiple-choice.

To assess environmental contamination at the SGU SVM Small Animal Clinic, samples were obtained once a week in the late afternoon from September to November 2018. Samples were collected from animal contact surfaces and human contact surfaces in the outpatient and inpatient areas of the Small Animal Clinic as shown in Supplementary File 2. A single swab (Dryswab™, MWE, Corsham, Wiltshire, UK) was used to take specimens from the animal contact surfaces in a particular area and another swab was used for taking specimens from human contact surfaces. For example, in the consultation room, one swab was used for all the animal contact surfaces and another swab for all the human contact surfaces.

### 2.2 Culture, isolation and identification of SP

Specimens were kept in a cool box during sampling and transport, and cultured within 48 h of collection at the SGU SVM Bacteriology laboratory. Each specimen was placed into 3 ml of tryptic soy broth (TSB; BBL Microbiology Systems, Cockeysville, MD, USA) containing 2.5% sodium chloride and incubated at 37 °C for 18–24 h. Enriched broths were streaked onto mannitol salt agar (MSA) plates (Difco Laboratories, Detroit, MI, USA) and incubated at 37 °C for 24–48 h. Suspect *Staphylococcus* species were identified based on morphologic features and mannitol fermentation (yellow colonies for mannitol fermenters; and pink/red colonies for mannitol non-fermenters). An isolated colony of suspected *Staphylococcus* was sub-cultured onto blood agar plate and incubated at 37 °C for 18–24 h. The MSA plates that lacked suspect colonies were incubated for additional 24 h before they were discarded. Round and raised medium sized colonies with beta hemolysis on blood agar plates were presumptive for *S. aureus, S. pseudintermedius, S. intermedius, S. schleiferi, S. delphini* or *S. lutrae*. An isolated colony per sample was picked, inoculated into 10% skimmed milk broth and stored at −20°C.

Presumptive *Staphylococcus* isolates were plated onto blood agar plates (BAPs) and a single colony from a plate was inoculated onto brain heart infusion (BHI) agar slants and shipped to co-author MA Davis's laboratory in compliance with a Centers for Disease Control and Prevention import permit. Presumptive staphylococci isolates were identified by polymerase chain reaction (PCR) (24) at the Davis lab and/or by matrix-assisted laser desorption/ionization time of flight (MALDI-TOF) mass spectrometry at the Washington Animal Disease Diagnostic Laboratory (WADDL, Pullman, WA, USA). The DNA for the PCR test was prepared by the boiled cell lysate method, and the PCR test was performed using primer pairs pse-F2 (5′-TRGGCAGTAGGATTCGTTAA3′), and pse-R5 (5′-CTTTTGTGCTYCMTTTTGG3′) targeting the thermonuclease gene (*nuc*). The run conditions were an initial cycle of 95 °C for 2 min, followed by 30 cycles of 95 °C for 30 s, 56 °C for 35 s, 72 °C for 1 min and a final extension at 72 °C for 2 min.

### 2.3 Antimicrobial susceptibility testing and *mecA* PCR

Susceptibility testing was performed against 10 antimicrobials: ampicillin (10 μg), chloramphenicol (30 μg), ciprofloxacin (5 μg), clindamycin (2 μg), erythromycin (15 μg), gentamicin (10 μg), oxacillin (1 μg), tetracycline (30 μg), trimethoprim/sulfamethoxazole (1.25/23.75 μg) and vancomycin (30 μg) using the Kirby Bauer disk diffusion assay (25) according to Clinical and Laboratory Standard Institute (CLSI) guidelines (26). The following quality control organisms were used: *S. aureus* ATCC 25923, *Pseudomonas aeruginosa* ATCC 27853, *Enterococcus faecalis* ATCC 29212 and *E. coli* ATCC 25922. The CLSI breakpoints for antimicrobial disk diffusion susceptibility tests were used to categorize each isolate as resistant or susceptible to antimicrobials tested. Isolates with intermediate resistance were classified as susceptible. Combined resistance was evaluated by concatenating individual resistance to the antimicrobials tested. Multidrug resistance (MDR) was defined as resistance to three or more antimicrobial classes.

The MRSP isolates were identified by screening all isolates for *mecA* gene using PCR as previously described (27, 28). Briefly, DNA was prepared by the boiled cell lysate method, and PCR was performed using forward primer 5′-ATACTTAGTTCTTTAGCGAT-3′ and reverse primer 5′-GATAGCAGTTATATTTCTA-3′. The run conditions were an initial cycle of 95 °C for 3 min, followed by 30 cycles of 96 °C for 30 s, 49 °C for 30 s, 72 °C for 30 s and a final extension at 72 °C for 5 min. The PCR products were visualized with agarose gel electrophoresis using 1.5% gel stained with GelRed^®^ (Biotium, Hayward, CA, USA). Reference strains *S. aureus* ATCC 25923 and *S. aureus* ATCC 33591 were used as controls.

### 2.4 Multi-locus variable number of tandem repeat analysis (MLVA) multiplex PCR

For the MLVA multiplex PCR, isolates were streaked onto Columbia blood agar plates (Hardy Diagnostics, Santa Maria, CA, USA) and incubated at 37 °C overnight. A single colony was picked and inoculated into BHI broth (Hardy Diagnostics, Santa Maria, CA, USA) and incubated at 37 °C overnight. Then, 2 ml of inoculated broth was centrifuged at 4,800 × g for 5 min, then washed with 1 × Phosphate-Buffered-Saline (PBS; Fisher Scientific, Waltham, MA, USA) and heated to 100 °C for 15 min. Then the sample was centrifuged at 8,300 × g for 10 min and the supernatant was transferred into a 1.5 ml tube and stored at −20 °C until used for PCR.

This multiplex PCR assay was developed in house at Washington State University (Supplementary File 3). The assay targets eight variable numbers of tandem repeats (VNTR) loci. The multiplex PCR uses eight primer pairs run in two reactions. Reactions 1 and 2 each contained four primer pairs at the concentrations listed. Both reactions used Qiagen multiplex master mix kit (Qiagen, Hilden, Germany), and 1 μl of bacterial DNA extract in a total volume of 10 μl. The PCR run conditions were as follows: an initial denaturation at 95 °C for 15 min, 30 cycles of amplification at 95 °C for 90 s, 55 °C for 90 s, and 72 °C for 90 s, followed by an elongation step of 72 °C for 10 min. The PCR products were diluted 1:20 with water, and 1 ml of the diluent was mixed with 0.5 μl of 1,200 LIZ size standard and formamide (Applied Biosystems, Waltham, MA, USA). The mixture was denatured by incubation at 95 °C for 5 min and capillary electrophoresis was conducted using a 3,730 DNA analyzer (Applied Biosystems).

The electropherograms were analyzed using GeneMarker V2.7.0 genotyping software (Softgenetics LLC, State College, PA, USA). The fragment size at each VNTR locus was used to determine the allele number as follows: (fragment length–flanking sequences length)/repeat length (29). For each isolate, the alleles at each of the eight VNTR loci were concatenated to form a MLVA profile. Thereafter, unique MLVA profiles were assigned MLVA types. The goeBURST algorithm in Phyloviz 2.0 software was used to construct minimum spanning trees and to visualize genotypic relationships among isolates from the different dog categories and environmental samples (30).

### 2.5 Statistical analysis

Statistical analysis was performed using R software version 4.2.0, Vienna, Austria (31). Differences in SP carriage between Grenadian-born dogs, and foreign-born dogs were compared using a two-way chi-square test of homogeneity (R function prop.test) or Fisher's exact test (R function fisher.test) and *p*-values < 0.05 were considered significant. Similarly, differences in antimicrobial resistance frequency among SP from Grenadian-born dogs, and foreign-born dogs were compared. Questionnaire data were analyzed using a generalized linear model to establish association between MRSP infection (binomial response variable) and several risk factors (explanatory variable) which included age, sex, antimicrobial use, hospitalization, dog health according to owner, and country/region of birth. The models were fitted using R function glm with a binomial family and logit link function. The forward step-up selection was used to select explanatory variables for inclusion in the final model starting with the variable that contributes the most to the response variable, and the cutoff *p*-value was 0.20.

## 3 Results

### 3.1 Questionnaire responses and analysis

The owners of 86 dogs responded and agreed to enroll their dogs in the study. Questionnaires were administered to dog owners and epidemiological data were obtained on 86 dogs. Complete data were obtained from 85 dogs and a summary is provided in Table 1. Most dogs were foreign-born (68.2%) with 50.6% from North America (predominantly USA, Canada, Puerto Rico), and 16.5% from European countries (Croatia, Holland, Hungary, Slovakia, United Kingdom), while 31.8% of the dogs were Grenadian-born. Most dogs were over 3 years old (74.1%), socialized with dogs from other households on a weekly basis (70.2%) and had visited a veterinary clinic or hospital within 6 months of sampling for preventive care or treatment (78.6%). Most dogs were reported by their owners to be in very good or excellent health condition (81.2%). However, approximately 60% of the dogs were either identified as having a skin condition at the time of sampling or owner-reported as having a skin condition within the past 6 months. About 25% of the dogs had received antimicrobials within 6 months of sampling and 6% were receiving antimicrobials at the time of sampling.

**Table 1:** Epidemiological data obtained from questionnaire administered to dog owners in Grenada in 2018.

**Variable**	**No. dogs**	**%**
**Country of birth/origin (*****N*** = **85)**		
Europe	14	16.5
Grenada	27	31.8
North America	43	50.6
Asia	1	1.2
**Sex (*****N*** = **85)**		
Male	44	51.8
Female	41	48.2
**Age (*****N*** = **85)**		
≤ 1 year old	5	5.9
1–2 years old	17	20.0
3–4 years old	29	34.1
>4 years old	34	40.0
**Owner reported health of dog (*****N*** = **85)**		
Fair	8	9.4
Good	8	9.4
Very good	36	42.4
Excellent	33	38.8
**Skin allergies or infection in past 6 months (*****N*** = **85)**		
Yes	51	60.0
No	34	40.0
**Antimicrobial use in last 6 months (*****N*** = **84)**		
Yes	21	24.7
No	63	74.1
**Antimicrobial use during sampling (*****N*** = **85)**		
Yes	5	5.9
No	80	94.1
**Hospital visit in last 6 months (*****N*** = **84)**		
Yes	66	77.6
No	18	21.2
**Hospitalization (*****N*** = **84)**		
Yes	8	9.4
No	76	89.4
**Socialize with other dogs (*****N*** = **85)**		
Yes	59	69.4
No	26	30.6
**Number dogs in household (*****N*** = **84)**		
Single	48	56.5
Multiple	38	44.7

N, number of dogs.

Further epidemiological analysis was performed on 55 dogs from whom SP was isolated. Risk factors for MRSP occurrence were determined by univariable analysis. The association of each of several variables on the occurrence of MRSP were evaluated and antimicrobial use in the past 6 months was the only variable associated with MRSP infection at 5% level of significance (Table 2). The other variables evaluated (sex, age, owner perceived health of dog, presence of skin condition, socializing with dogs from other households, whether a dog had visited a veterinary clinic/hospital in the past 6 months, region of origin) were not statistically significantly associated with MRSP occurrence. Multivariable models were not built because only one variable (antimicrobial use in the last 6 months) was significantly associated with MRSP infection.

**Table 2:** Simple univariable analysis to determine risk factors for the occurrence of MRSP in 55 dogs with epidemiological data and from whom SP was isolated in 2018.

**Variable**	***N***	***N* dogs with MRSP**	**% dogs with MRSP**	**Odds ratio**	**95% CI**	***p*-Value**
**Sex**						
Male	30	5	16.7	1.47	0.3–7.8	0.6263
Female	25	3	12.0			
**Age**						
≤ 1–2 years	12	1	8.3			
>2–4 years	20	5	25.0	3.7	0.5–75.6	0.2648
>4 years	23	2	8.7	1.04	0.09–24.0	0.971
**Has skin condition**						
Yes	34	4	11.8	0.67	0.15–3.1	0.46045
No	21	4	19.0			
**Antimicrobial use in last 6 months**						
Yes	14	6	42.9	14.6	2.8–113.4	0.003
No	41	2	4.9			
**Antimicrobial use during sampling**						
Yes	5	3	60.0	13.5	1.8–124.2	0.0113
No	50	5	10.0			
**Hospitalization**						
Yes	6	2	33.3	3.5	0.4–22.6	0.196
No	49	6	12.2			
**Socialize with other dogs**						
Yes	35	3	8.6	0.28	0.05–1.3	0.1104
No	20	5	25.0			
**Number dogs in household**						
Single	30	4	13.3	1.5	0.33–8.2	0.5862
Multiple	26	4	15.4			
**Dog health according to owner**						
Fair	5	2	40.0			
Good	6	1	16.7	0.29	0.01–4.5	0.3985
Very good	26	4	15.4	0.27	0.03–2.6	0.2214
Excellent	18	1	5.6	0.088	0.003–1.19	0.0776
**Country/region of birth**						
Grenada	14	3	21.4			
Europe	8	4	50.0	2.33	0.4–14.6	0.3373
North America	25	1	4.0	0.19	0.01–1.6	0.1626

N, number of dogs; CI, confidence interval; MRSP, methicillin resistant Staphylococcus pseudintermedius.

### 3.2 Detection of SP and MRSP in dogs and environmental samples

At least two samples (oral and perianal) were collected from each dog and additional samples were collected from dogs with skin or ear lesions. A total of 195 samples were obtained and comprised of 85 oral swabs, 85 perianal swabs and 25 skin/ear lesion swabs. Overall, SP was isolated from 73 out of 195 of samples (37.4%), and MRSP was isolated from 10 out of 195 samples (5.1 %). Details of SP and MRSP detection from the different anatomic sites is shown in Table 3. For example, among the 25 skin/ear lesion samples, SP was detected in 24% of the samples and MRSP was detected in one skin lesion (4%). The prevalence of SP in dogs was based on detection of SP from at least one anatomic site. The overall prevalence of SP was 64.7% and there was no difference in prevalence between Grenadian-born dogs and foreign-born dogs (Table 4). The overall prevalence of MRSP was 9.4% and there was no statistical difference in MRSP prevalence between Grenadian-born dogs and foreign-born dogs.

**Table 3:** Frequency of *S. pseudintermedius* in different anatomic sites in dogs in Grenada, 2018.

**Specimen type**	***N* SP positive**	**%**	**No. MRSP positive**	**%**
Oral swab (*N* = 85)	38	44.7	6	7.1
Perianal swab (*N* = 85)	29	34.1	3	3.5
Skin lesion swab (*N* = 25)	6	24.0	1	4.0
Total (*N* = 195)	73	37.4	10	5.1

N, number of dogs; SP, Staphylococcus pseudintermedius; MRSP, methicillin resistant Staphylococcus pseudintermedius.

**Table 4:** Prevalence of *S. pseudintermedius* in Grenadian and foreign dogs, 2018.

**Dog category**	***N* of dogs sampled**	***N* of dogs with SP**	**Prevalence of SP (%)**	***N* of dogs with MRSP**	**Prevalence of MRSP (%)**
Grenadian-born	27	17	63.0	3	11.1
Foreign-born	58	38	65.5	5	8.6
Total	85	55	64.7	8	9.4

N, number of dogs; SP, Staphylococcus pseudintermedius; MRSP, methicillin resistant Staphylococcus pseudintermedius.

A total of 42 environmental samples were obtained from animal contact surfaces in 6 areas of the SGU Small Animal Clinic and 42 samples were obtained from human contact surfaces from 6 areas as listed in Table 5. *Staphylococcus pseudintermedius* was isolated from both animal and from human contact surfaces in the consultation room; whereas SP was not detected in any of the human or animal contact surfaces in the anesthesia room or surgical suite. SP was isolated from human contact surfaces on the workstation and laboratory and from animal weighing scales. Overall, SP was isolated from 10 out of the 84 (11.9%) environmental samples. One isolate was MRSP and it was obtained from animal contact surfaces in the consultation room (Table 5).

**Table 5:** Frequency of *S. pseudintermedius* isolated from environmental surfaces at the St. George's University, Small Animal Clinic, Grenada, 2018.

**Area**	Animal contact surfaces			Human contact surfaces		
	***N*** **samples**	***N*** **positive**	**%**	***N*** **samples**	***N*** **positive**	**%**
Consultation room	7	3	42.9	7	1	14.3
Treatment room	7	2	28.6	7	0	0.0
Anesthesia room	7	0	0.0	7	0	0.0
Surgical suite	7	0	0.0	7	0	0.0
Outpatient hallway	7	1	14.3	NA	NA	NA
Inpatient hallway	7	1	14.3	NA	NA	NA
Work station	NA	NA	NA	7	1	14.3
Laboratory	NA	NA	NA	7	1	14.3
Total	42	7	16.7	42	3	7.1

N, number of samples; NA: not applicable.

### 3.3 Antimicrobial resistance of SP isolates

One isolate per positive sample was selected and a total of 83 SP isolates were available for further analysis. The frequency and percentage of SP isolates resistant to individual antimicrobials is shown in Table 6. In general, the most frequent resistance in SP isolates was to ampicillin followed by tetracycline regardless of whether they were from foreign-born dogs or Grenadian-born dogs. The AMR levels in SP from foreign-born dogs were generally similar to those found in Grenadian-born dogs (Table 6).

**Table 6:** Antimicrobial resistance in *S. pseudintermedius* isolated from dogs in Grenada in 2018, and from animal and human contact surfaces at the Saint George's University Small Animal Clinic, Grenada.

**Antibiotic**	SP (***n*** = 50) from foreign-born dogs		SP (***n*** = 23) from Grenadian-born dogs		Total SP (***n*** = 73) from dogs		SP (***n*** = 10) from evironmental surfaces at the SAC	
	**No. resistant**	**%**	**No. resistant**	**%**	**No. resistant**	**%**	**No. resistant**	**%**
Ampicillin	32	64.0	17	73.9	49	67.1	2	20.0
Tetracycline	19	38.0	15	65.2	34	46.6	3	30.0
Erythromycin	10	20.0	8	34.8	18	24.7	1	10.0
Trimethoprim/sulfamethoxazole	6	12.0	7	30.4	13	17.8	1	10.0
Clindamycin	4	8.0	8	34.8	12	16.4	0	0.0
Oxacillin	9	18.0	2	8.7	11	15.1	1	10.0
Fluoroquinolone	5	10.0	7	30.4	12	16.4	1	10.0
Gentamicin	0	0.0	0	0.0	0	0.0	1	10.0
Chloramphenicol	6	12.0	1	4.3	7	9.6	1	10.0
Vancomycin	0	0.0	1	4.3	1	1.4	0	0.0
Pansuceptible	10	20.0	4	17.4	14	19.2	6	60.0

SP, Staphylococcus pseudintermedius; SAC, Small Animal Clinic.

### 3.4 MLVA genotypes and relationships

**Figure 1:**
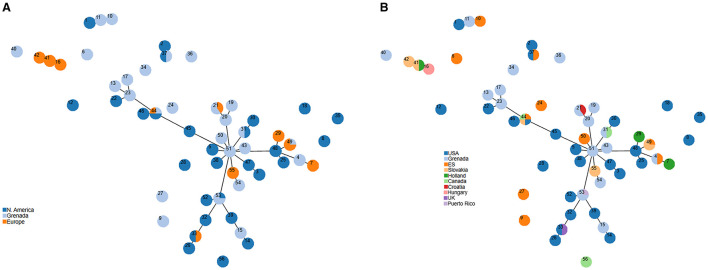
Minimum spanning tree of 56 MLVA genotypes (labeled 1–56) observed in 83 Staphylococcus pseudintermedius isolates collected in 2018 from foreign dogs (*n* = 50), Grenadian dogs (*n* = 23), and environmental samples (*n* = 10). Each circle represents a MLVA genotype and the size is proportional to the number of isolates. Genotypes with single locus variants are connected by solid lines and form a cluster complex and smaller clusters with closely related genotypes form around founder genotypes. It shows the relationship between MLVA genotypes and region of origin **(A)**, or country of birth **(B)**.

The MLVA genotyping was performed on 83 isolates and included isolates from foreign dogs (*n* = 50), Grenadian dogs (*n* = 23) and environmental samples (*n* = 10). A total of 56 unique MLVA genotypes were identified, and genotypic relationships was determined by constructing a minimum spanning tree at the single-locus-variant level. A MLVA cluster was formed if 7 of the 8 VNTR loci were identical. The analysis resolved the 56 genotypes into one main cluster complex where genotypes with single-locus-variants were depicted as connected by solid lines. In addition, there were two tripletons, one doubleton, and 8 unconnected singletons. The main cluster complex comprised smaller clusters with closely related genotypes connected to founder genotypes 23, 48, 51 and 53. Some genotypes were exclusive to SP obtained from dogs from certain countries or regions, while some genotypes were shared across countries or regions (Figures 1A, B). For example, genotypes #21, #31 and #53 were shared by isolates from Grenadian dogs and foreign dogs from Croatia, Canada, and Puerto Rico, while genotype #44 was shared by isolates from environmental sample, and dogs from Canada, Slovakia, and USA. Conversely, genotypes #16, #41, and #42 formed a tripleton that was exclusive to SP obtained from Europe-born dogs from Holland, Hungary and Slovakia.

### 3.5 Description of MRSP isolates

The concordance between phenotype (determined by oxacillin susceptibility assay) and genotype (determined by presence of *mecA* gene) was 89.2%. Oxacillin resistance was observed in 14.5% (12/83) of the SP isolates; however, *mecA* gene was detected in 8 isolates and these were termed oxacillin-resistant MRSP (OR-MRSP). Four oxacillin resistant isolates lacked the *mecA* gene and were classified as MSSP. Of the 71 oxacillin-susceptible isolates, *mecA* gene was present in three isolates and these were termed oxacillin-susceptible MRSP (OS-MRSP).

A description of the 11 MRSP isolates is provided in Table 7. Most of the MRSP isolates had MDR profiles and were resistant to at least six classes of antimicrobials. MRSP isolates from three dogs from the same dwelling belonged to MLVA genotype #49 and were resistant to six or seven classes of antimicrobials. These dogs came from Slovakia (Europe) and arrived in Grenada in June 2016 and June 2017, and had visited the SGU Small Animal Clinic within 6 months of sampling. Notably, MRSP isolate #ES26 from animal contact surfaces at the SGU Small Animal Clinic was the same strain as that found in these dogs. Two MRSP isolates from a dog that came from Croatia (Europe) and arrived in Grenada in January 2017 shared MLVA genotype #21 with an isolate from a Grenadian-born dog with travel history to the USA, and another isolate from a Grenadian-born dog with no travel history outside the island. The MRSP isolates belonging to genotype #21 came from dogs that had received antimicrobials within 6 months of sampling. The dog from Croatia was receiving antimicrobials at the time of sampling, had visited the SGU Small Animal Clinic on multiple occasions and had been hospitalized. Grenadian-born dog D068 with no travel history had also visited and been hospitalized at the same Small Animal Clinic. An MRSP isolate from Grenadian-born dog D088 had unique MLVA genotype #23 but the same resistance pattern to isolates with MLVA genotypes #21 and #49. However, an OS-MRSP isolate from a USA-born dog showed single resistance to ampicillin and belonged to singleton MLVA genotype #35.

**Table 7:** Description of methicillin resistant *Staphylococcus pseudintermedius* isolates (*n* = 11) obtained from dogs in Grenada in 2018, and animal contact surface at the SGU Small Animal Clinic, Grenada.

**Dog ID or source**	**Isolate ID**	**Country of birth**	**Travel history or first arrival in Grenada**	**Visit to SAC in past 6 months**	**Hospitalized at SAC in the past 6 months**	**Antimicrobial use in the past 6 months**	**Phenotypic resistance profile**	**MLVA type**	**Other comments**
D010	D010-P	USA	Arrived in August 2017	Yes	No	Clavamox, clindamycin	AMP	35	Urinary tract infection
D047	D047-O	Slovakia	Arrived in June 2018	Yes	No		AMP-CHL-FQR-CLI-ERY-SXT-OXA-TET	49	Dogs D047, D049 and D058 live together
D049	D049-O	Slovakia	Arrived in June 2018	Yes	No	Cefpodoxine	AMP-CHL-FQR-CLI-ERY-SXT-OXA-TET	49	Dogs D047, D049 and D058 live together
D058	D058-O	Slovakia	Arrived in June 2016	Yes	No		AMP-FQR-CLI-ERY-SXT-OXA-TET	49	Dogs D047, D049 and D058 live together
D063	D063-O	Croatia	Arrived in January 2017	Yes many times	Yes	Clindamycin, trimethylsulfate	AMP-FQR-CLI-ERY-SXT-OXA-TET	21	On antibiotics during sampling
	D063-P						AMP-FQR-CLI-ERY-SXT-OXA-TET	21	
D068	D068-O	Grenada	No travel outside Grenada	Yes	Yes	Yes but no details provided	AMP-FQR-CLI-ERY-SXT-TET	21	Skin condition
	D068-P						AMP-CHL-FQR-CLI-ERY-SXT-TET	21	
D087	D087-O	Grenada	Traveled to USA in 2018	Yes	No	Cefodosine	AMP-FQR-CLI-ERY-OXA-TET	21	Urinary tract infection
D088	D088-L	Grenada	No travel outside Grenada	Yes	No	Cephalexin	AMP-FQR-CLI-ERY-SXT-OXA-TET	23	Fair health, has skin condition
Environment	ES26	NA	NA	NA	NA	NA	AMP-CHL-FQR-CLI-ERY-SXT-OXA-TET	49	Animal contact surface in consultation room

NA, not applicable; AMP, ampicillin; CHL, chloramphenicol; CLI, clindamycin; FQR, fluoroquinolone; ERY, erythromycin; OXA, oxacillin; TET, tetracycline; SXT, trimethoprim/sulfamethoxazole; N, number; MLVA, multiple-locus variable number of tandem repeats, SGU; Saint George's University; SAC, Small Animal Clinic.

## 4 Discussion

This is the first report of the occurrence of MRSP, and resistance to chloramphenicol and fluoroquinolone among SP isolates from dogs in Grenada, and contrasts with a 2014 study that only found methicillin susceptible staphylococci in dogs with pyoderma; among the 66 pyoderma cases in that study, 28 had SP and none were methicillin resistant (20). Furthermore, results from the study reported here showed a higher frequency of resistance to most of the antimicrobials tested compared to the 2014 study. Consistent with other reports, most of the MRSP isolates from this study showed resistance to multiple antimicrobials, and detection of MRSP was associated with antimicrobial use. These observations among MRSP isolates have important implications for animal health due to limited treatment options for clinical MRSP infections, such as pyoderma, otitis, urinary tract infections and surgical wound complications (1, 15, 16, 32). There are also implications for public health due to several reports of zoonotic infections with MDR MRSP strains (33–35).

The hypothesis that MRSP might be introduced to Grenadian-born dogs by foreign-born dogs is not fully supported based on epidemiological, genotyping and AMR findings from the present study. Our findings are consistent with reports that international movement of dogs can disseminate MRSP. For example, similar MRSP strains based on MLVA genotype and AMR pattern were observed in a Croatian-born dog that arrived in Grenada in 2017, a Grenadian-born dog with travel history to the USA, and a Grenadian-born dog with no travel history. The Croatian-born dog (D063) was reported to be in fair health, was receiving antimicrobials at the time of sampling, visited the Small Animal Clinic multiple times and was hospitalized. Grenadian-born dog #D068 had also visited the same Small Animal Clinic and had been hospitalized, and transmission might have occurred during hospitalization or a hospital visit. It's also plausible that Grenadian-born born dog #D087 might have been exposed to MRSP while traveling in the USA, or while in Grenada. International movement of dogs for trade or recreation is reported to contribute to dissemination of MRSP strains (17, 22). Dogs are known to carry MRSP strains for several months providing lengthy periods of time for transmissions to occur (1, 36, 37).

Furthermore, finding a similar MRSP strain (MLVA type#49 and AMR pattern AMP-CHL-FQR-CLI-ERY-SXT-OXA-TET) in foreign-born dogs from Slovakia and arrival in Grenada in 2016 and 2017, and on animal contact surfaces in a consultation room at the Small Animal Clinic suggests the dogs may have contaminated the hospital environment during visits. The MRSP strain was detected in dogs in spring 2018 while detection at the Small Animal Clinic occurred in fall 2018, and given these dogs had visited the same clinic previously, it is plausible infected dogs could have contaminated the environment and resulted in persistence for several months. Transmissions of MRSP between dogs and the environment have been documented (38, 39).

The prevalence of SP in healthy dogs in this Grenada study (64.7%) is comparable to previous reports from other countries around the world; for example, 64% in Brazil (40), 53% in Poland (41), 25%−65.9% in the USA (41) and 37%−92% in various locations (42). Differences in SP prevalence can be attributed to various factors such as geographical location, diagnostic methods and anatomic sites sampled. In this study, samples were collected from more than one anatomic site and this approach increases overall diagnostic sensitivity (43). Simultaneous sampling from multiple anatomic sites (pharynx, perineum, corner of the mouth and wounds when available) is recommended for MRSP detection (36). The overall frequency of MRSP infection in clinically healthy dogs in this study is 9.4% and MRSP was detected in 5.1% of specimens. These results are comparable to MRSP prevalence of 2.6% in healthy dogs in Norway (44), 2% in USA, 1.5% in Slovenia (45), and 7% in healthy dogs in Canada (15). Similarly, a systematic review of nasal MRSP carriage in healthy dogs reported pooled MRSP prevalence of 3.1% (46). Conversely, a study in New Zealand (47) and in Poland (41) did not detect MRSP in healthy dogs. The prevalence of MRSP in foreign-born dogs and Grenadian-born dogs in this study were not statistically different. Multiple factors affect MRSP occurrence such as antimicrobial use, frequent hospital visits, environmental sanitation and dog movements (7, 12, 16, 22). The continuous international movement of SGU faculty, staff and students and their dogs between other countries and Grenada presents an ongoing risk for MRSP dissemination. Also, a high proportion of dog owners reported their dogs socialized on weekly basis with dogs from other households and such interactions provide opportunities for pathogen spread.

Whereas 60% of dogs were reported by their owners to have a skin condition within a period of 6 months, superficial dermatitis was observed in 28% of dogs during physical examinations. The discrepancy between dog-owner reported skin conditions and observations made during physical examination could be due to subjective responses by the owners or the skin conditions could have resolved by the time of examination. *Staphylococcus pseudintermius* was isolated in 24% of the skin lesion specimens collected, and MRSP was detected in only one skin lesion. Other causes of the observed superficial dermatitis could be other staphylococcal species, allergies, fleas, mites, fungal or bacterial infections.

Contamination and persistence of MRSP and other bacteria in hospital environments resulting in nosocomial infections and disease outbreaks is well-established (7, 8). Detection of SP in animal contact surfaces in the consultation and treatment rooms, weighing scales and equipment that are frequently used highlights the challenges, and importance of adequate cleaning and/or disinfection practices. Similarly, detection of SP in human contact surfaces in the consultation room, laboratory and computer workstations is expected given these surfaces are frequently touched by staff and might get contaminated due to inadequate hand hygiene practices. Occurrence of similar genotypes and AMR patterns in the environment and in dogs that visited the Small Animal Clinic suggests possible transmission events. Absence of SP detection in the anesthesia room and surgical suite implies good infection prevention and control in these areas. Guidelines for prevention of SP transmission in veterinary settings include hand hygiene, appropriate use of personal protective equipment, cleaning, disinfection and surveillance (1).

Methicillin resistance status of isolates in this study was determined by detection of the *mecA* gene. A study that evaluated concordance between antibiotic phenotypes and genotypes found 85% agreement between phenotypic oxacillin resistance and genotypic methicillin resistance (48). Another study reported good correlation between phenotype and genotype when an oxacillin breakpoint of ≤ 17 mm is used in the disk diffusion assay (49). The latter breakpoint was used in the current study and the concordance is similar to what has been reported. One OS-MRSP isolate in this study had single resistance to ampicillin, and this finding is consistent with reports that single resistance to benzyl penicillin is common among OS-MSRP isolates (50).

Dogs in this study were not randomly selected but rather based on targeted recruitment and voluntary participation of dog owners. Also, the sample size of the Grenadian-born dogs was small (*n* = 27). Hence, the samples may not be representative of the dog population in Grenada and the study findings may be biased. Nonetheless, the study findings provide useful insights about SP and MRSP occurrence, AMR levels and patterns, and MRSP dissemination via international dog movements. Also, this study used MLVA to assess genotypic relationships among SP isolates; hence, it was not possible to compare our results with global clones that use an MLST scheme and whole genome sequence analysis. Nonetheless, 56 unique MLVA genotypes were found among 83 isolates which is consistent with reports that SP populations are genetically highly diverse (17, 22). Future investigation of SP isolate population structure and antimicrobial resistance using whole genome sequence and MLST analysis would provide in-depth insights about transmission pathways, and how the strains in Grenada relate to global strains (5, 16, 22).

## 5 Conclusion

This is the first report of MRSP occurrence, and resistance to fluoroquinolone and chloramphenicol among SP isolates from dogs in Grenada and contrasts a 2014 study that only found methicillin susceptible staphylococci in dogs with pyoderma. Higher levels of SP resistance to ampicillin, clindamycin and erythromycin were observed in this study compared to the 2014 study. Analysis of MLVA genotypes, AMR patterns and epidemiological data suggests international movement of dogs could be consistent with the observed findings, but the source of MRSP emergence in dogs in Grenada could not be determined based on the present findings. Detection of similar MRSP strains in foreign-born dogs and environmental samples at the Small Animal Clinic might suggest occurrence of environmental contamination and nosocomial transmissions. This highlights the importance of ongoing targeted AMR surveillance in dogs at high risk for MRSP and surveillance in the veterinary clinic environment. Knowledge of antimicrobial susceptibility profiles of circulating SP strains is crucial for choosing antimicrobials that are effective for managing clinical infections. Furthermore, implementing appropriate infection prevention and control in the veterinary clinic needs to be emphasized. Future studies using whole genome sequencing and MLST analysis would be invaluable for comparison of SP isolates in Grenada to global clones.

## Data Availability

The original contributions presented in the study are included in the article/Supplementary material, further inquiries can be directed to the corresponding author.

## Supplementary material

Supplementary File 1 Questionnaire on demographics, health, travel history and social interactions of dogs in Grenada.

Supplementary File 2 Description of animal and human contact surfaces sampled at the SGU, SVM, Small Animal Clinic in 2018.

Supplementary File 3 MLVA multiplex PCR primers and procedures for *Staphylococcus pseudintermedius*.
