# Supplementary material for: Emergence of methicillin resistant Staphylococcus pseudintermedius in dogs sampled in 2018 in the island nation of Grenada, West Indies
Source: Front Vet Sci. 2026 Mar 18;13:1761713. doi: 10.3389/fvets.2026.1761713 (PMC13041561; doi:10.3389/fvets.2026.1761713)
Supplement: Supplementary File 1 — Questionnaire on demographics, health, travel history and social interactions of dogs in Grenada. [file Data_Sheet_1.pdf]

Research Study Investigating Transmission of *Staphylococcus pseudintermedius* in Dogs in Grenada

The purpose of this questionnaire is to collect information on the demographics, health, travel, and social interactions of dogs in Grenada. This information will be used to assess the risk factors for transmission of *Staphylococcus pseudintermedius* in dogs in Grenada.

1. Date

	DD	MM	YYYY
Date		/	/

2. Identification code (to be assigned by researcher)

3. What is the age of your dog?

- ○ 1 - 12 months old
- ○ 1 - 2 years old
- ○ 2+ - 4 years old
- ○ Over 4 years

4. What is your dog's gender?

- ○ Female
- ○ Male

5. Has your dog been spayed or neutered?

- ○ Yes
- ○ No

6. What is the breed of your dog?

- ○ Grenada Pothound

Other (please specify)

7. In which state or country was your dog born?

- ◯ Grenada

Other (please specify)

[ ]

8. If your dog was born in Grenada, has your dog traveled outside Grenada?

- ◯ Yes
- ◯ No

9. If your dog was not born in Grenada, when is the first time your dog visited Grenada?
9. If your dog has traveled outside Grenada, when is the last time your dog returned to Grenada?

[ ]

10. In the past year, how many months has your dog spent in Grenada

[ ]

11. How would you rate your dog's health in the last 6 months?

- ◯ Excellent
- ◯ Very good
- ◯ Good
- ◯ Fair
- ◯ Poor

12. Has your dog visited a veterinary clinic or hospital in the last 6 months?

- ◯ No
- ◯ Yes (please specify or name the clinic or hospital)

[ ]

13. In the last 6 months, was your dog hospitalized?

- ◯ Yes
- ◯ No

14. In the last 6 months, did your dog have any of the following skin conditions? Please indicate all answers that apply.

- ◯ Itchy skin
- ◯ Scratching affected skin
- ◯ Licking affected skin
- ◯ Chewing affecting skin
- ◯ Rubbing skin against an object
- ◯ Hair loss
- ◯ Open sores
- ◯ Skin infection with pus
- ◯ Other (please specify)

15. In the last 6 months, has your dog been given medications?

- ◯ No
- ◯ Yes (please specify if antibiotics, analgesics, anti-inflammatory, dewormer, or other category)

16. Is your dog currently on antibiotics?

- ◯ No
- ◯ Yes (please give the name of the antibiotic)

17. Has your dog ever experienced difficulty in responding to antibiotics to treat skin or other conditions?

- ◯ Yes
- ◯ No

18. How many dogs do you have in Grenada?

19. Does your dog intermingle, play, or socialize with other dogs in the neighborhood?

- ◯ No
- ◯ Yes (please specify how often)

20. Swab collection site (to be filled by researcher)

- ◯ Oral cavity
- ◯ Nasal cavity
- ◯ Perineum/rectum

Other (please specify)

[ ]
