# Supplementary material for: Emergence of methicillin resistant Staphylococcus pseudintermedius in dogs sampled in 2018 in the island nation of Grenada, West Indies
Source: Front Vet Sci. 2026 Mar 18;13:1761713. doi: 10.3389/fvets.2026.1761713 (PMC13041561; doi:10.3389/fvets.2026.1761713)
Supplement: Supplementary File 2 — Description of animal and human contact surfaces sampled at the SGU, SVM, Small Animal Clinic in 2018. [file Data_Sheet_2.pdf]

Supplementary file 2 Description of animal and human contact surfaces sampled at the SGU SVM Small Animal Clinic in 2018

**Area**	**Animal contact surfaces**	**Human contact surfaces**
Consultation room	Top of examination table, stethoscope bell/diaphragm, otoscope speculum, ophthalmoscope	2 computer keyboards & mice, stethoscope, otoscope & ophthalmoscope handles, microscope knobs, door knobs, water tap handles, drawer/cabinet handles, hand sanitizer container surface
Outpatient hallway	Weighing scale surfaces where animals are placed/stand	
Inpatient hallway	Weighing scale surfaces where animal is placed/stands	
Work station		4 computer keyboards & mice
Treatment room	3 examination tables, stethoscope bell/diaphragm, muzzles, collars, leashes, hair clipper blades	Telephone handle/dial, stethoscope, otoscope & ophthalmoscope handles, drawer/cabinet handles, infusion pump
Laboratory		Fridge handles, door knobs, machine keypads, keyboards, drawer/cabinet handles
Anesthesia room	2 examination tables, stretcher, face masks	Door knobs, drawer/cabinet handles
Surgical suite	Surgical table	Door knobs, infusion pump
