# Supplementary material for: Emergence of methicillin resistant Staphylococcus pseudintermedius in dogs sampled in 2018 in the island nation of Grenada, West Indies
Source: Front Vet Sci. 2026 Mar 18;13:1761713. doi: 10.3389/fvets.2026.1761713 (PMC13041561; doi:10.3389/fvets.2026.1761713)
Supplement: Supplementary File 3 — MLVA multiplex PCR primers and procedures for Staphylococcus pseudintermedius. [file Data_Sheet_3.pdf]

Bacterial growth and DNA extraction

Staphylococcus pseudintermedius isolates stored in -80ºC were grown onto Columbia blood agar plates (Hardy Diagnostics, CA USA) at 37ºC overnight. One colony was picked and placed in BHI broth (Hardy, CA USA) and incubated at 37ºC with 180 rpm shaking overnight. Next 2 mL of bacterial broth was centrifuged at 4800 x g for 5 min and washed with 1 X PBS (Fisher Sci,), and heated to 100ºC for 15 min. Then centrifuged for 10 min at 8300 x g. The supernatant was transferred to a 1.5 mL tube and stored at -20ºC until used for PCR.

MLVA Primers

MLVA primers were developed using both Tandem Repeat Finder version 2.0 (https://tandem.bu.edu/cgi-bin/trdb/trdb.exe?taskid=1) and Geneious 11.0 using 10 genomes that we sequenced, and 4 that were publicly available. The different genomes were uploaded to the database and the repeats were screened within the Geneious software. All repeats were identified within each genome and then aligned using MAFFTv 7.388 to identify the repeat regions. Repeats that showed differences were selected to be turned into primers using Geneious Primer3 software. Each primer was tested as an individual and checked against many isolates to see if there were differences within the band sizes. We started with 13 primers and ended up with eight primers (regions) that showed differences with the repeat region. If the primers had difference, the primer was then moved into one of two multiplexes.

MLVA PCR

Two multiplexes were developed to perform the MLVA reaction. Reaction 1 contained VNTR_10FAM, VNTR_12NED, VNTR_6NED, and VNTR_8PET at the primer concentration listed below (Table 2). Reaction 2 contained VNTR_4PET, VNTR5_VIC, VNTR_7FAM, VNTR_11 at the concentration listed below (table 2). Both reactions used Qiagen Multiplex Master Mix Kit (Qiagen, Hilden, Germany) with 1 µl of supernatant from the bacterial extractions for a total a volume 10 µl. The fused the following PCR run conditions: initial denaturation (15 min at 95ºC), 30 cycles of amplification (90 s at 95ºC, 90 s at 55ºC, and 90 s at 72ºC) followed by elongation step of 10 min at 72ºC.

After the PCR run, samples were diluted into 1:20 in water. 1 mL of the diluted solution was mixed with 0.5 µl of 600 LIZ size standard (Applied Biosystems) and formamide (Applied Biosystems). The mixture was then denatured by incubation for 5 min at 95ºC and then run on an ABI 3730. The results were analyzed in GeneMarker V2.7.0 software.

VNTR	Region	Repeat size	Start	Stop
VNTR 6	Dru-region	40bp	36608	37107
VNTR 8	NON-coding	28bp	932914	933169
VNTR 10	Class A beta-lactamase	12bp	2630421	2630828
VNTR 12	Cyclic-di-AMP phosphodiesterase GdpP CDS	12 bp	18296	18413
VNTR 4	Zinc ABC transporter, substrate-binding lipoprotein AdcA CDS	18bp	291166	291526
VNTR 5	Translation initiation factor 2 CDS	18bp	1629034	1629474
VNTR 7	Beta-lactamase regulatory sensor-transducer BlaR1 CDS	31bp	2631419	2631968
VNTR 11	hypothetical protein CDS	6bp	2392234	2392414

VNTR	DYE	Primer Forward	Primer Reverse	RXN	Conc.
VNTR6	NED	GGAGTTAATCTACGTCTCATCTCA	TCGGTTGTAATTTTCATTTTTATACGC	1	0.32
VNTR 8	PET	AAGCGTTGGTTAGTTGAAGGA	ACATCGTTGGTGACTCCTTT	1	0.2
VNTR 10	FAM	ACGTTGCTTTTTCGATTGAT	GCTGATAAAAGTGGTCAAGC	1	0.4
VNTR 12	NED	TGTCATGCTCATTATCAGTGT	CATTGTTTTGACTGCACGAT	1	0.2
VNTR 4	PET	TGAGCCGACACAAAAAGATA	TGGATCATAACCCCCATGA	2	0.2
VNTR 5	VIC	ACCGTAATCAGATGCAACTA	GAAGATGACCAAGTGAAAGC	2	0.2
VNTR 7	FAM	CTTTGGCATGTGAACTGTTT	AAACTATCCAAATAACTGTGCA	2	0.4
VNTR 11	FAM	TAATATCATCAACTGCCGCA	CCTTTGAGTAACGCGATATC	2	0.2
